# Research progress on the relationship between chronic prostatitis/chronic pelvic pain syndrome and the microbiota of the reproductive system

**DOI:** 10.3389/fcimb.2024.1417276

**Published:** 2024-06-21

**Authors:** Wei-Jie Song, Xin-Yi Liu, Le-Ye He

**Affiliations:** ^1^ Department of Urology, Central South University, The Third Xiangya Hospital, Changsha, Hunan, China; ^2^ Sexual Health Research Center, Central South University, The Third Xiangya Hospital, Changsha, Hunan, China; ^3^ The Fifth Department of Gynecology, Hunan Provincial Maternal and Child Health Care Hospital, Changsha, Hunan, China

**Keywords:** chronic prostatitis/chronic pelvic pain syndrome, reproductive system microorganisms, clinical diagnosis, treatment, research progress

## Abstract

Chronic prostatitis/chronic pelvic pain syndrome (CP/CPPS) is a common pelvic pain syndrome in males, seriously affecting patients’ quality of life. For a long time, CP/CPPS has been considered a complex and variable disease, and its pathogenesis remains incompletely understood. Currently, CP/CPPS is believed to be a group of diseases characterized by pelvic pain or discomfort, urinary abnormalities, and other symptoms, each with its unique etiology, clinical characteristics, and outcomes, likely resulting from the action of pathogens or (and) certain non-infectious factors. Traditionally, CP/CPPS was thought to be unrelated to bacterial infections. However, in recent years, with the development of microbiology and the advancement of high-throughput sequencing technology, an increasing number of studies have suggested that microorganisms in the reproductive system may play an important role in the pathogenesis of CP/CPPS. The unique characteristics of CP/CPPS, such as its refractory nature and tendency to recur, may be closely related to the microbiota and their biological functions in the reproductive system. The relationship between CP/CPPS and reproductive system microorganisms is one of the current hot topics in microbiology and urology, receiving considerable attention from scholars in recent years and making a series of new advances. Through this review, we will comprehensively explore the relationship between CP/CPPS and reproductive system microorganisms, and look forward to future research directions, aiming to provide new ideas and methods for clinical diagnosis and treatment, thereby improving the treatment outcomes and quality of life of CP/CPPS patients.

## Introduction

The National Institutes of Health (NIH) classifies prostatitis into four types, with Type III being CP/CPPS (Chronic Prostatitis/Chronic Pelvic Pain Syndrome). This type is the most common, accounting for over 90% of all cases. It further divides into Type IIIA and IIIB based on abnormal increases in white blood cells in prostatic massage fluid, post-massage urine, or semen (Krieger et al., 1999). Diagnosis of CP/CPPS relies on medical history, physical examination, and laboratory tests. Detailed history-taking and physical examination help exclude other conditions with similar symptoms, such as infection or tumors. Urine analysis, prostatic massage fluid examination, and imaging studies aid in ruling out other diseases and assisting in the classification of prostatitis (Polackwich and Shoskes, 2016; Song et al., 2023b). Questionnaire tools like the National Institutes of Health Chronic Prostatitis Symptom Index (NIH-CPSI) assess the severity of symptoms by evaluating aspects like pain/discomfort, urinary frequency, urgency, and voiding difficulties (Pena et al., 2021). By integrating these diagnostic methods, a comprehensive assessment of symptoms and signs can be made to ultimately diagnose CP/CPPS.

The pathogenesis of CP/CPPS remains unclear, with complex and contentious etiology. It may result from a single initiating factor or involve multiple factors acting together, with some playing key roles and interacting (Mehik et al., 2003; Pontari and Ruggieri, 2004; Peng et al., 2009; Li et al., 2022). Firstly, pathogen infections may be associated with CP/CPPS onset. Although conventional bacterial tests fail to isolate pathogens, certain bacteria may evade culture or have low absolute abundance below the culture threshold (Song et al., 2023a). Specific pathogens like Chlamydia trachomatis, Ureaplasma urealyticum, trichomonads, fungi, and viruses have been implicated (Pontari and Ruggieri, 2004). Secondly, urinary dysfunction is a significant factor. Overactive urethral sphincter contraction can cause bladder outlet obstruction and residual urine formation, leading to urine reflux into the prostate. This not only potentially introduces pathogens but also directly stimulates the prostate, triggering sterile chemical prostatitis and resulting in clinical symptoms like abnormal urination or pelvic area pain (Mehik et al., 2003). Additionally, psychological factors play a crucial role. Long-term CP/CPPS patients often exhibit significant psychological problems and personality changes. Adverse psychological factors can lead to autonomic nervous system dysfunction, causing urethral neuro-muscular dysfunction and subsequently pelvic pain and voiding dysfunction (Li et al., 2022; Song et al., 2023b). Other factors contributing to CP/CPPS include neuroendocrine factors, immune response abnormalities, oxidative stress theories, pelvic-related disorders, and lower urinary tract epithelial dysfunction. All these factors may play a role in CP/CPPS onset (Peng et al., 2009). Overall, the pathogenesis of CP/CPPS is highly complex, necessitating further research to unravel its mysteries.

## The diversity and functionality of microbiota in the reproductive system

Both male and female urethras contain normal microbial communities, and whether the prostate is in a sterile state remains controversial. The sterile collection of biological samples and obtaining these samples from healthy males present current challenges in studying the internal conditions of the prostate. Therefore, further research is needed to investigate the diversity and functionality of the microbial community in the male reproductive system. In the past, it was widely believed that the male urethra and prostate were sterile. However, recent studies have found that these areas also harbor rich microbial communities, which play a crucial role in maintaining the health of the male reproductive system and may help prevent the invasion and infection of pathogens by maintaining a normal microbial balance (Feng and Liu, 2022).

Microbial diversity refers to the presence and relative abundance of different types of microorganisms. Studies have revealed that microbial communities in the male urethra and prostate consist of various microorganisms including bacteria, fungi, viruses, and others (Nickel, 2017; Feng et al., 2019). Each individual’s microbial composition is unique and influenced by factors such as individual differences, environmental factors, and lifestyle habits. Through the study of microbial communities, scientists have gradually elucidated the richness and diversity of microorganisms in the male reproductive system (Wise and Shteynshlyuger, 2006; Borges et al., 2019; He et al., 2021).

The functions of microorganisms in the male reproductive system mainly include: 1. Antimicrobial activity: Microorganisms inhibit the growth and spread of pathogens by occupying ecological niches in the reproductive tract. They compete for nutrients, produce antimicrobial substances, thereby reducing the risk of infection caused by pathogens (Zuber et al., 2023). 2. Immunomodulation: Microorganisms interact with the host immune system to regulate the balance of immune responses. They can promote the activation of immune cells, enhance immune defense capabilities, and help the host resist the invasion of pathogenic microorganisms (Colella et al., 2023). 3. Maintenance of mucosal health: Microorganisms have a significant impact on the health of the male reproductive tract mucosa. They maintain the integrity of the mucosal barrier, preventing the invasion of harmful substances. Additionally, microorganisms participate in regulating mucosal immune function, maintaining the normal state of mucosal tissue (Brotman et al., 2014). 4. Impact on germ cell function: Imbalance in microbial communities may affect the development and function of male germ cells. Existing research suggests that microorganisms can indirectly affect sperm quality and quantity by secreting metabolites, influencing hormone levels, and other pathways, thereby impacting reproductive health (Chen et al., 2023).

## The association between CP/CPPS and microbiota of the reproductive system

The association between CP/CPPS (Chronic Prostatitis/Chronic Pelvic Pain Syndrome) and microbiota of the reproductive system has been a subject of considerable interest. Although the exact cause of CP/CPPS remains unclear and conventional microbiological tests often fail to identify pathogenic bacteria, an increasing body of research suggests that microbiota may play a significant role in the pathogenesis of CP/CPPS. Multiple studies have indicated the presence of various microorganisms, including bacteria, fungi, and viruses, in prostatic massage fluid and urine samples from CP/CPPS patients (Shoskes et al., 2016; Mändar et al., 2017; Choi et al., 2020; Wu et al., 2020; Song et al., 2023a). The association between microbiota and CP/CPPS may involve several potential pathogenic mechanisms. Firstly, microbial infections may directly cause inflammation reactions in the prostate and pelvic tissues, leading to pain and discomfort (Miyake et al., 2022). Secondly, microbial metabolites and bacterial toxins may affect the nervous and immune systems, resulting in increased pain transmission and immune dysfunction (Mayerhofer et al., 2017). In addition to direct pathophysiological effects, microorganisms may also indirectly participate in the onset and development of CP/CPPS by influencing the host’s microbial balance, neuroendocrine system, and immune regulation (Mjaess et al., 2023).

Although an increasing number of studies support the role of microbiota in CP/CPPS, many questions remain to be further researched and addressed. Firstly, under the influence of inflammation, the prostatic ducts may become occluded. Most existing studies have used semen, prostatic massage fluid, or post-massage urine as biological samples, which may not provide complete and accurate information about the internal conditions of the prostate. Additionally, the relative importance of microbial infections in the pathogenesis of CP/CPPS, the differential roles of different types of microorganisms, and the mechanisms of interaction between microbiota and the host are still unclear. Therefore, further research will contribute to a better understanding of the association between CP/CPPS and microbiota, providing theoretical foundations and clinical guidance for future diagnostic, therapeutic, and preventive strategies.

## Discussion

CP is a chronic disease that significantly affects the quality of life in males. In the past, it was commonly regarded as a type of prostatic infectious disease (Ikeuchi, 1988). Earlier research primarily focused on revealing the relationship between bacterial infection and the onset of CP. However, in the late 20th century, understanding of this disease remained relatively vague, with its clinical manifestations and etiology unclear. Research regarding CP and the microbiota of the reproductive system was just beginning to explore the connection between the two and their potential roles (Features of etiologic structure and factors of persistence of bacteria isolated during infection of lower urinary tract and chronic bacterial prostatitis, 2012).

Early studies on CP and reproductive system microbiota relied mainly on traditional bacterial culture methods to test samples such as urine, urethral secretions, and prostatic massage fluid from patients to determine the presence of bacterial infection. The core idea of these methods was to confirm infection by culturing potential bacteria in the laboratory. However, early research results were inconsistent. Some studies found bacteria in samples from some CP patients, while others did not find any bacteria (Meares and Stamey, 1968; Thin and Simmons, 1983; Greenberg et al., 1985; Shortliffe, 1985; Meares, 1987). This inconsistency led researchers to question whether bacterial infection was the sole or primary cause of CP. Although early studies failed to reach a consensus on the relationship between bacterial infection and CP, they provided important insights, driving a deeper understanding of the etiology and treatment of CP. It was not until 1995 that the NIH first proposed the classification and definition of CP. The NIH classified chronic prostatitis into Type I (acute bacterial prostatitis), Type II (chronic bacterial prostatitis), and Type III (chronic prostatitis/chronic pelvic pain syndrome, CP/CPPS). Among them, Type III CP/CPPS was defined as patients experiencing chronic pelvic pain but without detection of bacterial infection in samples such as prostatic fluid and urine (Krieger et al., 1999). This definition clarified the classification and diagnostic criteria for CP/CPPS, distinguishing it between non-bacterial CP/CPPS and bacterial acute and chronic prostatitis. Subsequently, research and clinical practice in the medical field gradually developed based on this foundation.

Although the NIH’s classification and definition provided a common framework for the research and treatment of CP/CPPS, there are still controversies and uncertainties regarding the etiology and mechanisms of non-bacterial CP/CPPS. With the development of metagenomic techniques, researchers began using methods such as 16S rRNA gene sequencing to study changes in the microbial communities of CP/CPPS patients. These studies found that the composition of the reproductive system microbiota might be related to the occurrence and development of the disease even in patients without obvious infectious symptoms (Shoskes et al., 2016; Mändar et al., 2017; Choi et al., 2020; Wu et al., 2020; Song et al., 2023a). This provided new clues for further exploring the role of microbiota in CP/CPPS.

Recent studies have shown that metagenomic research plays an important role in revealing the pathogenesis of CP/CPPS. By analyzing the microbial composition of patient samples from the reproductive system, researchers can gain a more comprehensive understanding of the microbial spectrum associated with CP/CPPS. Microbes are not merely present; their interaction with the host plays a crucial role in disease development. Each person’s microbial composition is unique and can be influenced by factors such as individual differences, environmental factors, and lifestyle habits. Existing studies have shown that the reproductive system microbiota of CP/CPPS patients are diverse and functionally potent. Studies on samples such as prostatic massage fluid, semen, and urine from CP/CPPS patients have found enrichment of specific microbes in these samples (Shoskes et al., 2016; Mändar et al., 2017; Choi et al., 2020; Wu et al., 2020; Song et al., 2023a). Compared to healthy individuals, CP/CPPS patients show significant differences in the abundance and structure of reproductive system microbiota, and these differentially abundant microbes possess certain biological functions that may affect inflammation levels and pain perception through mechanisms such as metabolite production and immune response modulation, thus influencing the occurrence and development of CP/CPPS (Choi et al., 2020; Song et al., 2023a).

Understanding the characteristics and functions of the microbial community in CP/CPPS patients allows targeted adjustments to restore the microbial balance of the prostate and pelvic cavity, thereby alleviating inflammatory responses and pain perception. This provides new ideas and strategies for individualized treatment, aiming to improve the quality of life of CP/CPPS patients. However, basic research on the microbiology of CP/CPPS is still in its infancy and requires further exploration. Current research mainly relies on the analysis of samples such as prostatic massage fluid, semen, and urine, which may only reflect part of the situation. Future research could consider collecting a more diverse range of samples to gain a more comprehensive understanding of the microbiological characteristics of CP/CPPS.

In terms of clinical trials and treatment strategies, some studies have begun to explore microbial intervention strategies for the treatment of related diseases. Antibiotic treatment targeting microbes is a common intervention strategy. Specific antibiotics can selectively kill or inhibit the microbes causing the disease. In addition to antibiotic treatment, probiotic intervention is also a treatment strategy worth considering. Beneficial microbes for host health can treat related diseases by regulating the host’s immune system and metabolic functions. Furthermore, microbial transplantation is an emerging treatment strategy. Microbial transplantation involves transferring the microbial community of healthy individuals into patients to improve their microbial composition and function (Hempel et al., 2012; Magri et al., 2019; Antushevich, 2020). Although these experimental treatments have not yet been applied to CP/CPPS patients, they may provide important evidence for the future development of clinical treatment strategies for CP/CPPS.

We believe that microbial prediction and individualized treatment targeting microbes are promising research directions for the diagnosis and treatment of CP/CPPS in the future. By analyzing large-scale microbial data, there is a possibility to establish predictive models to help identify high-risk individuals early and take corresponding preventive measures to reduce the incidence of CP/CPPS. Combining personalized treatment plans based on microbial information will also become an important part of treatment, maximizing treatment effectiveness and minimizing patient suffering. Although microbes show great potential in the field of CP/CPPS, there are still some challenges. Establishing a more comprehensive microbial database and standardized experimental procedures will be the focus of future research to ensure the reliability and repeatability of research results. In-depth study of the interaction mechanisms between microbes and host immune systems, nervous systems, and others will help comprehensively understand the impact of microbes on the occurrence and development of CP/CPPS, but this is also a challenging task. The conduct of clinical trials and validation of treatment effects require interdisciplinary collaboration and support from large-scale clinical samples.

## Conclusion

In conclusion, an increasing number of researchers are focusing on the relationship between CP/CPPS and microbiota of the reproductive system. Microbiota of the reproductive system play a significant role in the onset and development of CP/CPPS, and the imbalance of the reproductive tract microbiota may affect the overall health of the reproductive system. Certain specific microorganisms may be closely associated with the pathogenesis of CP/CPPS. Targeted therapy against microorganisms presents a promising strategy for treating CP/CPPS. Compared to traditional treatment methods, personalized treatment strategies theoretically offer more specificity and efficacy. Future research needs to further explore the specific mechanisms of microbiota in the onset and development of CP/CPPS and conduct large-scale clinical trials to provide new insights and strategies for the prevention and treatment of CP/CPPS.

## Author contributions

WS: Conceptualization, Supervision, Writing – original draft, Writing – review & editing. XL: Supervision, Writing – original draft. LH: Supervision, Validation, Writing – review & editing.
